# Breast cancer disease classification using fuzzy-ID3 algorithm with FUZZYDBD method: automatic fuzzy database definition

**DOI:** 10.7717/peerj-cs.427

**Published:** 2021-05-04

**Authors:** Nur Farahaina Idris, Mohd Arfian Ismail

**Affiliations:** Faculty of Computing, College of Computing and Applied Sciences, Universiti Malaysia Pahang, Pekan, Pahang, Malaysia

**Keywords:** Fuzzy, ID3 algorithm, FID3 algorithm, Fuzzy decision tree, FUZZYDBD, Breast cancer, Classification, Fuzzification

## Abstract

Breast cancer becomes the second major cause of death among women cancer patients worldwide. Based on research conducted in 2019, there are approximately 250,000 women across the United States diagnosed with invasive breast cancer each year. The prevention of breast cancer remains a challenge in the current world as the growth of breast cancer cells is a multistep process that involves multiple cell types. Early diagnosis and detection of breast cancer are among the greatest approaches to preventing cancer from spreading and increasing the survival rate. For more accurate and fast detection of breast cancer disease, automatic diagnostic methods are applied to conduct the breast cancer diagnosis. This paper proposed the fuzzy-ID3 (FID3) algorithm, a fuzzy decision tree as the classification method in breast cancer detection. This study aims to resolve the limitation of an existing method, ID3 algorithm that unable to classify the continuous-valued data and increase the classification accuracy of the decision tree. FID3 algorithm combined the fuzzy system and decision tree techniques with ID3 algorithm as the decision tree learning. FUZZYDBD method, an automatic fuzzy database definition method, would be used to design the fuzzy database for fuzzification of data in the FID3 algorithm. It was used to generate a predefined fuzzy database before the generation of the fuzzy rule base. The fuzzified dataset was applied in FID3 algorithm, which is the fuzzy version of the ID3 algorithm. The inference system of FID3 algorithm is simple with direct extraction of rules from generated tree to determine the classes for the new input instances. This study also analysed the results using three breast cancer datasets: WBCD (Original), WDBC (Diagnostic) and Coimbra. Furthermore, the comparison of FID3 algorithm with the existing methods is conducted to verify the proposed method’s capability and performance. This study identified that the combination of FID3 algorithm with FUZZYDBD method is reliable, robust and managed to perform well in breast cancer classification.

## Introduction

Breast cancer is the most aggressive type of cancers suffered by women worldwide and becomes the second leading cause of death among women cancer patients (Lee & Han, 2014). Each year, approximately 250,000 women across the United States have been diagnosed with invasive breast cancer (Watkins, 2019). The primary cause of breast cancer disease is mainly related to patients inheriting the genetic mutations in their genes (Majeed et al., 2014). Breast cancer can cause rapid metastasis to occur which leads the primary tumour to vigorously spreading the breast cancer cells to distant organs like the bone, liver, lung and brain (Sree Kumar, Radhakrishnan & Cheong, 2010). The metastatic traits of breast cancer are mostly accountable for the high incurability rate (Sun et al., 2017). Although advancements in breast cancer treatment lead to a decrement in breast cancer mortality rates in all age groups, the young age remains a high-risk factor and has a low survival rate (Lee & Han, 2014). Early diagnosis of breast cancer patients is substantial for averting the rapid progression of breast cancer aside from the evolution of preventative procedures (Sun et al., 2017).

Diagnosis of breast cancer can be made manually by the physician, but it will take a longer period of time and must be very intricate for the physician to implement the classification (Khuriwal & Mishra, 2018). The incompleteness of relevant data can also lead to human errors in diagnosis (Zaitseva et al., 2020). Thus, breast cancer detection through an intelligent system is vital in the medical field. Various methods can be applied for classification of breast cancer such as Neural Network, Support Vector Machine, KNN and decision tree (Khuriwal & Mishra, 2018; Kuo et al., 2008). This paper proposed a new version of the fuzzy-ID3 algorithm (FID3 algorithm) to improve breast cancer classification efficiency. This study’s primary purpose is to develop a method that can overcome the limitation of traditional ID3 algorithm that is unable to classify the continuous-valued data and also increase the classification performance. The ID3 algorithm, which is the most commonly used decision tree learning, treats the continuous-valued attributes as discrete attributes with many possible values (Al-Ibrahim, 2011; Patil., Agrawal & Baviskar, 2015). It is designed to only handle discrete and categorical data. The FID3 algorithm implements the data fuzzification and linguistic variable replacement process to handle the continuous-valued data. The advantage of this method is that it has high comprehensibility and interpretability of a decision tree and can cope with inaccurate and uncertain information in fuzzy representation.

Nevertheless, for the FID3 algorithm that conducts data fuzzification, the fuzzy database must be defined. The automatic definition of the fuzzy database using a genetic algorithm and clonal selection algorithms has high computational cost and complexity (Cintra, Camargo & Martin, 2009; Cintra, 2012). Thus, this paper implements the FUZZYDBD method to design the fuzzy database in the FID3 algorithm as an approach to producing a fast and effective system. The inference process of the FID3 algorithm is also made simple by using inductive reasoning like the traditional ID3 algorithm. The testing and verification process is implemented to validate the performance of the method. The rest of this paper was organised as follows, starting from Materials and Methods, Results, Discussion and lastly the Conclusion.

## Materials and Methods

### Fuzzy system

**Figure 1:**
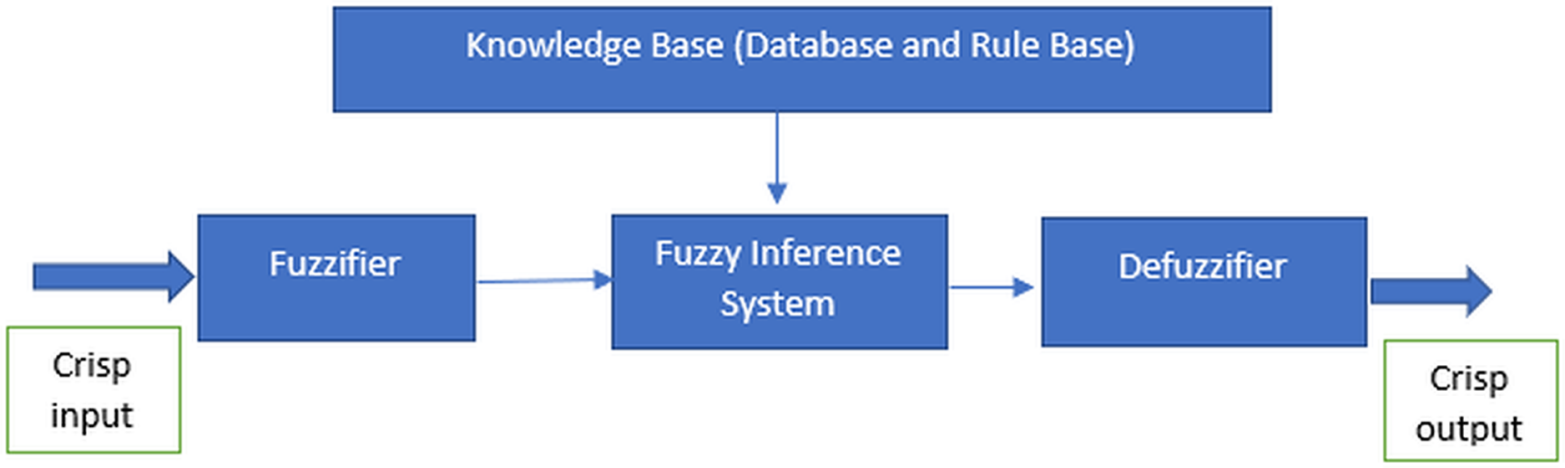
Features of the fuzzy system.

The fuzzy system is derived from the concept of fuzzy logic proposed by Zadeh in 1965, which essentially is a precise logic of imprecision and approximate reasoning (Zadeh, 2008). It functions in the form of logical variables which the values are within the range of 0 and 1. It is usually being implemented to handle the imprecision problems in the data using the fuzzy set theory (Cintra, Monard & Camargo, 2013a). According to Ben-mubarak et al. (2012) and Thaker & Nagori (2018), there are four most important features to implement the fuzzy system which are the fuzzifier, inference engine, fuzzy base or knowledge base and defuzzifier as illustrated in Fig. 1. The features are required for the processes in the fuzzy system.

**Figure 2:**
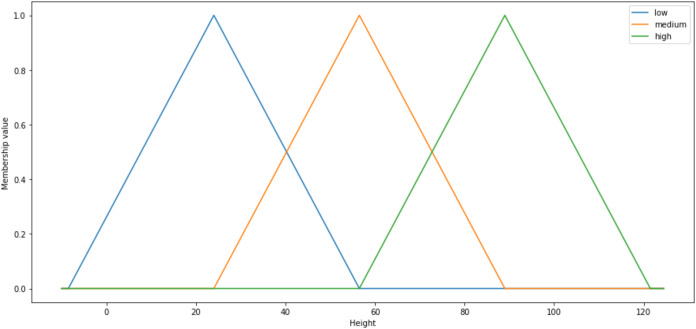
Shape of the triangular membership function.

The fuzzy system implementation is comprised of three essential processes which are fuzzification, inference engine and defuzzification. In order to conduct fuzzification via fuzzifier, fuzzy systems needed the granulation of the feature of the domain, which are the fuzzy sets and partitions (Cintra, Monard & Camargo, 2013a). The fuzzy sets and partitions would form the membership functions. Every fuzzy set is uniquely defined by a single distinct membership function (Dai, Gao & Dong, 2010). Thus, the particular membership functions are commonly symbolised as the labels of the respective fuzzy sets. Range of values correspond to each of the fuzzy set is also assigned to each input factor (Thaker & Nagori, 2018). Membership functions hold the degree of membership used to measure the grade of membership for the fuzzy sets. Figure 2 is given the example of triangular membership functions with three particular fuzzy sets labelled low (blue), medium (orange) and high (green):

The fuzzy system generally comprises of Rule-Based Fuzzy System that has two critical elements which are the knowledge base and an inference mechanism (Cintra et al., 2011c). The knowledge base consists of fuzzy rule base (FRB) that contains a set of fuzzy rules for the respective domains and fuzzy database which holds the definitions of the fuzzy sets involving the linguistic variables applied in FRB (Marcos Evandro Cintra & De Arruda Camargo, 2007). Meanwhile, the inference mechanism generates the outputs from the system using fuzzy reasoning. Then, the inference mechanism, which is also known as an inference engine, would utilise the fuzzy rules to map the input to output (Ahmadi et al., 2018). Inference engine would produce the most desirable consequents for each rule. Fuzzy parameters like rules and membership functions are codependent and essential in developing a fuzzy inference system (FIS) (Peña-Reyes & Sipper, 2001). The fuzzy rule can be expressed by:

$$IF(V1\;i\;IS\;A1\;i)\;AND\;(V2\;i\;IS\;A2\;i)\;AND\ldots (V_{j}\;i\;IS\;A_{j}\;i)\;THEN\;(CLASSi\;IS\;C\;i)$$

*V* has represented the linguistic variable, which is the attribute of the data. Meanwhile, *A* is the linguistic value, which is the value of the data. Then, *j* is the number of attributes in the data, while *i* is depicted as the number of rules. The most distinct keys in the fuzzy system are the use of linguistic variables, the interdependence of the variables through conditional rules and validation of complex interdependence using the fuzzy method (Zadeh, 1973). Use of linguistic variables in the fuzzy system means through the implementation of fuzzy the particular variables can be defined in natural language. Meanwhile, the interdependence of variables through conditional rules means linguistic variables in the antecedent section of fuzzy rules express as the attributes while the consequent section is the class. Validation of complex interdependence using fuzzy methods deems that the interdependence between class and linguistic variable can be validated with fuzzy logic.

Lastly, defuzzification is being implemented when the crisp values are required. In defuzzification, consequents can be aggregated to generate crisp output. It is a step to interchange the fuzzy output into crisp output using the fuzzy set and degree of membership which also known as membership value (Thaker & Nagori, 2018). The process of defuzzification takes place in defuzzifier (Saad & Wahyunggoro, 2010). There are many methods to execute defuzzification, such as the centre of gravity (COG), mean of maximum (MOM) and centre average methods (Masoum & Fuchs, 2008). The fuzzy system is a well-known classification algorithm in machine learning because of its simplicity but manage to produce high accuracy in classification (Surya et al., 2012).

The study performed by Ali & Mutlag (2018) conveys that the fuzzy method’s implementation is useful in detecting breast cancer as the accuracy reaches over 98%. The study made by Johra & Shuvo (2017) shows that the fuzzy logic model’s accuracy is 94.26% when applied with histopathology image dataset to classify benign and malignant cells in breast cancer tumours. Result of breast cancer thermogram classification by Schaefer et al. (2007) that also implemented the fuzzy method had achieved the diagnostic accuracy rate of 80%. High accuracies result based on the previous studies show the efficiency of the method in solving classification problems.

### Decision tree: ID3 algorithm

Decision tree (DT) is a well-known method in machine learning to implement classification. The advantage of using DT includes the high interpretability, scalability, and ability to illustrate in both graphic and text formation (Begenova & Avdeenko, 2018a). The most popular decision tree learning algorithm are ID3, C4.5 and CART algorithm. The decision tree learning algorithm that would be studied in this paper is ID3 algorithm as it is the most commonly implement learning algorithm at the moment (Chen, Luo & Mu, 2009; Liu & Wang, 2010; Luo, Chen & Zhang, 2010). Quinlan invented the ID3 algorithm that also known as Iterative Dichotomiser 3 in 1986 (Liu & Xie, 2010; Nijhawan, Madan & Dave, 2017). Theoretically, ID3 algorithm function based on recursive partitioning which the training data would undergo splitting to become subsets and the particular subsets become the partitions that depict the decision tree (Begenova & Avdeenko, 2018a; Wu et al., 2006). ID3 algorithm uses Shannon’s entropy and information gain as the attribute selection criteria (Wu et al., 2006).

The main element in the ID3 algorithm is the selection of the attributes for the tree by using the largest value of information gain. Information gain becomes the attribute selection criteria for the tree in order to choose the most qualified attribute for branching (Liu & Xie, 2010). The branching process would occur recursively until the tree achieves the termination conditions like all the attributes in the datasets being fully classified or all the balance instances has the same class. The ID3 algorithm can only be generated if the applied datasets have more than one class attribute (Wu et al., 2006). It will also produce rules for the class prediction and concurrently point out the respective class attributes (Teli & Kanikar, 2015). Generally, the algorithm utilises the top-down greedy approach to generate the decision tree. The significant aspect of the algorithm is that it would reduce the tree size using the quality measure and logical reasoning.

The research conducted by (Angayarkanni & Kamal, 2012) used MRI mammogram image dataset to test the performance and capability of the ID3 algorithm in the classification of breast cancer domain. The particular dataset consists of three class attributes, which are benign, malignant and normal. Results of the average accuracy of the ID3 algorithm is 99.9%. Meanwhile, the training time is over 0.03 s. It shows that method can achieve good classification result in short training time. The study made by Yang, Guo & Jin (2018), reveals that the algorithm can achieve the correct prediction accuracy over 90.56 % when tested with Wisconsin Breast Cancer Dataset (WBCD). Aside from that, the study implements by Jacob & Geetha Ramani (2012) depicts that the ID3 algorithm managed to perform even better than other classifiers such as Naïve Bayes and C-PLS in terms of classification accuracy when conducted with Wisconsin Prognostic Breast Cancer (WPBC). There are many advantages of implementing the ID3 algorithm as the DT learning algorithm, and the most significant advantage is that it takes short execution time (Chai & Wang, 2010; Idris & Ismail, 2020).

### Fuzzy decision tree

The fuzzy decision tree (FDT) is an extension of a decision tree (Zhai et al., 2018). The combination of both fuzzy and decision tree classifiers has an advantage in terms of handling the uncertainties and ambiguity data (Li, Jiang & Li, 2012; Wang et al., 2000). The application of data fuzzification is common practice to produce a robust model (Cintra, Monard & Camargo, 2013a). Many types of FDT available shows the high efficiency of the two combined classifiers (Umanol et al., 1994). The implementation of FDT commonly being be executed using general Shannon’s entropy or fuzzy entropy such as Luca-Termini and Kosko (Mitra, Konwar & Pal, 2002; Zhai et al., 2018). Cintra and Camargo originally invented a new method of FDT in 2010 using the combination of fuzzy and C4.5 algorithm call FUZZYDT algorithm (Cintra & Camargo, 2010; Cintra, 2012). FUZZYDT algorithm produces a fuzzy version of C4.5 algorithm as it still implements information gain, gain ratio and Shannon entropy for attribute selection criteria like traditional C4.5 algorithm (Cintra, Monard & Camargo, 2013b). Post pruning with the confidence interval of 25% customarily been applied to soar up the performance of the method but it also can be applied with pre pruning or without pruning (Cintra, Monard & Camargo, 2013b; Cintra et al., 2011b; Ribeiro, Camargo & Cintra, 2013). FUZZYDBD method conventionally had been used to determine the fuzzy set parameters in this method, while the classic and general fuzzy reasoning had been used to test the testing data (Cintra, 2012). It got error rates of 1.49% compared to C4.5 algorithm with 5.13% when tested with breast dataset.

Later, the fuzzy decision tree method was further studied by Begenova and Avdeenko using fuzzy and ID3 algorithm (Begenova & Avdeenko, 2018a). This method applies the approximate reasoning to the test the testing data, trapezoidal membership functions and bottom-up partitioning discretisation for distribution of fuzzy sets functions (Avdeenko, Makarova & Begenova, 2018; Begenova & Avdeenko, 2018a; Fajfer & Janikow, 2000). It acquired an accuracy of 95.65% with Iris dataset (Begenova & Avdeenko, 2018a). The distinct differences between the two versions of FDT are the selection of decision tree learning, type of reasonings and the fuzzy sets parameters for data fuzzification, especially the distribution of fuzzy sets and shape of membership functions. The reliability and efficiency of the fuzzy database definition method of FDT based on ID3 algorithm by Begenova and Avdeenko not empirically tested with breast cancer domain. The lacks of literature resources to explain the complex issues in existing FDT based on ID3 algorithm inspired this paper to study the new automatic fuzzy definition method for FDT that works well for breast cancer domain. The other notable approach of FDT is by Olaru and Wehenkel that developed a soft decision tree (SDT) that applies pruning, refitting and back fitting. This method's strategy is searching for the attribute and split location using crisp heuristics from the CART regression tree and implementing the fuzzification and labelling by explicit linear regression formulas. The back fitting and refitting process in this version of FDT is the tuning process which the refitting would optimise terminal nodes parameters, and back fitting would optimize all model-free parameters. The study finds out that SDT (back fitting) had a lower error rate of only 11.6% than CART and C4.5 algorithm, which were 19.5% and 19.2 respectively when tested with Omib dataset (Olaru & Wehenkel, 2003). Another approach like Tolerance Rough FDT used the degree of tolerance rough fuzzy dependency to select expanded attributes, Luca-Termini entropy to select optimal cut, and Kosko entropy for the termination condition got 98.19% with WDBC dataset (Zhai et al., 2018).

Fuzzy decision tree integrates a graphical representation of rules in tree form and fuzzy formation of data. The benefit for fuzzification of data is that the tree would be better in handling the continuous values attribute (Begenova & Avdeenko, 2018b). The traditional decision tree would split the data value into crisp intervals accordingly by minimising the entropy and maximising information gain which would lead to unnatural divisions and impacted the interpretability of the generated rules (Cintra, Monard & Camargo, 2013b). The other advantage of implement data fuzzification is the reuse of features or attributes. Traditional decision tree-like C4.5 algorithm can include the same attribute several times in one single rule, especially for continuous data with real values and range forms. It can lead to repetitive use of the same attribute and subdivision of the domain (Cintra, Monard & Camargo, 2013b). The issue concerning as it also reduces the interpretation of generated rules. FDT that apply fuzzification of training data is more robust and managed to overcome the issues in the classic decision tree (Cintra, 2012).

### FUZZYDBD method

The fuzzy automatic definition method is significant to develop a fuzzy database, and there are three elements involve to automatically defining the fuzzy database (Cintra, Camargo & Martin, 2009). Firstly, the automatic definition method can assist in determining the shape of the membership functions then, the number of fuzzy sets for each attribute in the domain and lastly the distribution of fuzzy sets for each attribute in the domain (Cintra, 2012). The fuzzy automatic definition method existed to enable the setup of fuzzy sets’ parameters in the fuzzy database more efficiently without burdening the domain experts. There are various existing methods can be applied for the definition of fuzzy databases in order to determine the number of fuzzy sets and tune the membership functions such as genetic algorithm, artificial neural network and fuzzy clustering algorithm (Aliev et al., 2011; Liao, Celmins & Hammell, 2003; Pulkkinen & Koivisto, 2010). Despite many methods exist, it is essential to highlight that many studies implement the definition number of fuzzy sets through empirical testing and just set the distribution of fuzzy sets evenly for the membership function because of the high complexity of the available methods and flexibility of fuzzy logic that can be adjusted the parameters to acquire better performance (Cintra, Camargo & Martin, 2009). Furthermore, there is a lack of consensus and guidelines on which existing methods can work the best for each application and domain (Cintra et al., 2011d).

**Figure 3:**
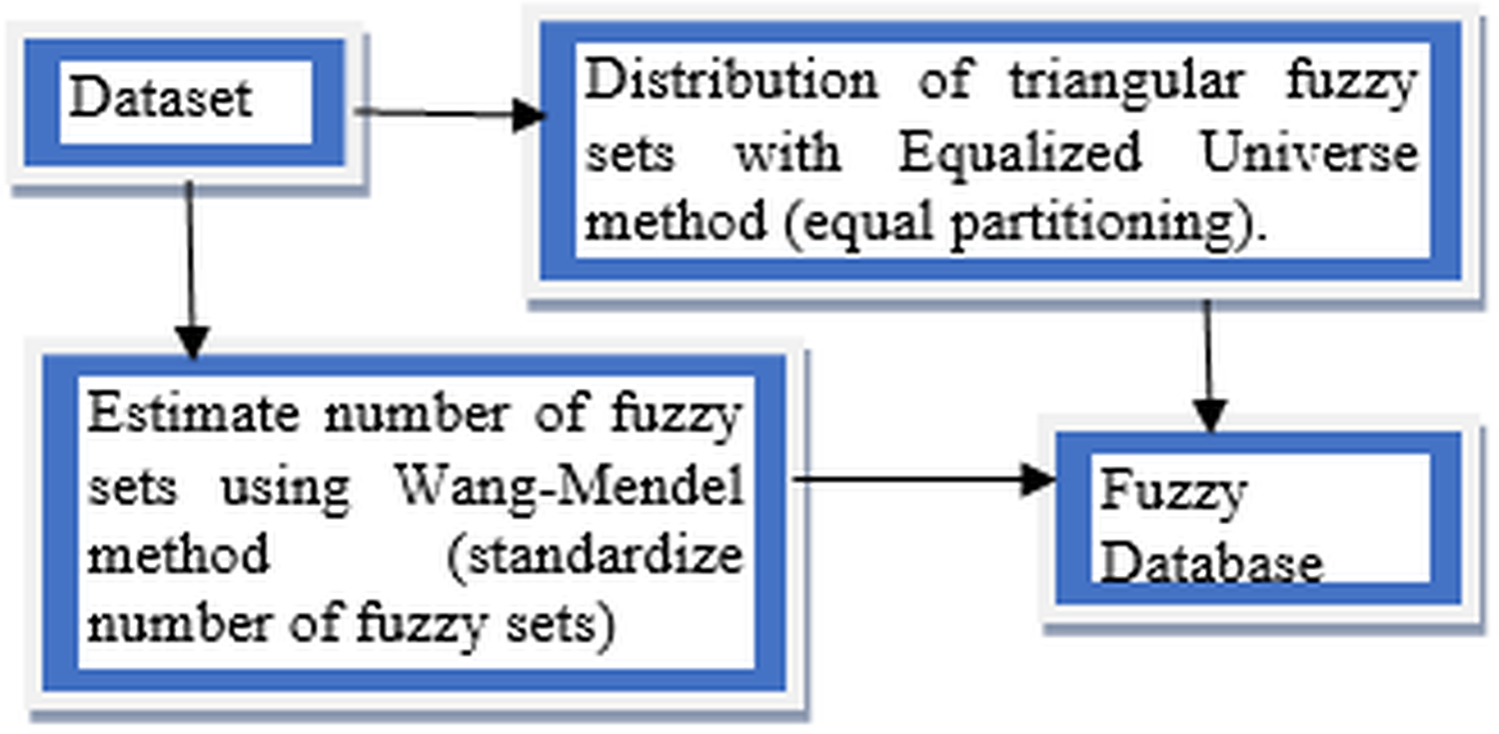
Approaches of FUZZYDBD Method.

This article conducts a further study on FUZZYDBD method that firstly proposed in Cintra, Camargo & Martin (2009) as it is a fast, simple and effective method for definition of the fuzzy database (Cintra, 2012). It also had been empirically tested in various domains, including breast cancer. FUZZYDBD method aggregates all the needed elements (ex. distribution of fuzzy sets) and resolves the issues with existing methods to define the fuzzy database (Cintra, Camargo & Martin, 2009; Cintra et al., 2011a, 2011d). According to Cintra (2012), the approaches of FUZZYDBD method including the definition of the number of fuzzy sets for all attributes using Wang Mendel method (Wang & Mendel, 1992), adoption of Equalized Universe method for the distribution of fuzzy sets for all attributes in the domains and application of triangle membership functions like in Fig. 3.

The number of fuzzy sets is standardised and same for all attributes in the datasets which the number of fuzzy sets can be between the range of 2 to 10 triangular membership functions. The best value between the range can be identified via empirical testing. The help of domain expert also can be used to define more suitable values of fuzzy sets for the attributes (Cintra, 2012; Cintra et al., 2014). The Wang Mendel method applies the same number of fuzzy sets, distribution and fuzzy sets shape to define all attributes in the collected dataset (Cintra, 2012). Although the range between 2 and 10 can be used to define the number of fuzzy sets, the values of 3, 5 and 7 are more commonly applied in the studies of FUZZYDBD method (Cintra et al., 2011b, 2014; Cintra & Neves, 2013; Cintra, 2012). Both values of 2 and 3 also are the best-estimated number of fuzzy sets for breast cancer dataset with the lowest error rate (Cintra, Camargo & Martin, 2009).

The Equalised Universe Method adopted by FUZZYDBD method was invented by Chen & Wang (1999). The method is applied the same width for each fuzzy set to produce an equal partitioning for the fuzzy sets in the attributes of the domain. The most maximum value of the respective attribute would be placed at the peak of most right triangular membership function while the most minimum value of the attribute would be placed at the peak of the most left triangular membership function. Thus, with this technique, the generated fuzzy values would not bound to has any error. This method is widely used in the literature (Cintra, 2012). The application of triangular equally partitioning membership functions implemented in FUZZYDBD with half overlap between the membership function ensures that no area has a membership degree more than 0.5. The most significant advantage of this method is that it is producing fuzzy databases that are very effortless and interpretable.

### Proposed method: FID3 algorithm

The decision tree is well-known as the method with low bias and high variance, and increasing the tree complexity will further decrease the bias and increase the variance (Olaru & Wehenkel, 2003). FID3 algorithm was developed to obtain a low complexity fuzzy decision tree that reduces the traditional decision tree’s high variance. The proposed method in this research, FID3 algorithm is inspired by FDT based on ID3 algorithm by Begenova & Avdeenko (2018a) that using information gain and Shannon’s entropy for attribute selection criteria like the traditional ID3 algorithm and also the method by Kumar, Varma & Sureka (2011) that apply fuzzification of the dataset. FID3 algorithm uses the ID3 algorithm as the classifier to handle the fuzzified data while applying the FUZZYDBD method to determine the fuzzy sets’ parameters used in the fuzzification process. Implementation of the inference system in FID3 algorithm, which uses all the rules extracted directly from the fuzzy decision tree is more interpretable and easier to understand than the existing method. FID3 algorithm computes the membership value for each input in the attributes and enumerates the confidence degree for every rule. The test data would automatically apply with the rules that have the highest compatibility degree with the input pattern to determine the class based on logical reasoning. The proposed method that applies fuzzification to the whole dataset favourably preserve the privacy of the patients as the precise data regarding the patients are concealed. Medical privacy is vital to maintain the security and confidentiality of patients’ records. The use for the linguistic variable is efficient, mainly when there is a coalition of support of the linguistic terms cover its entire domain which would effectively generate better accuracy and performance (Kumar, Varma & Sureka, 2011).

**Figure 4:**
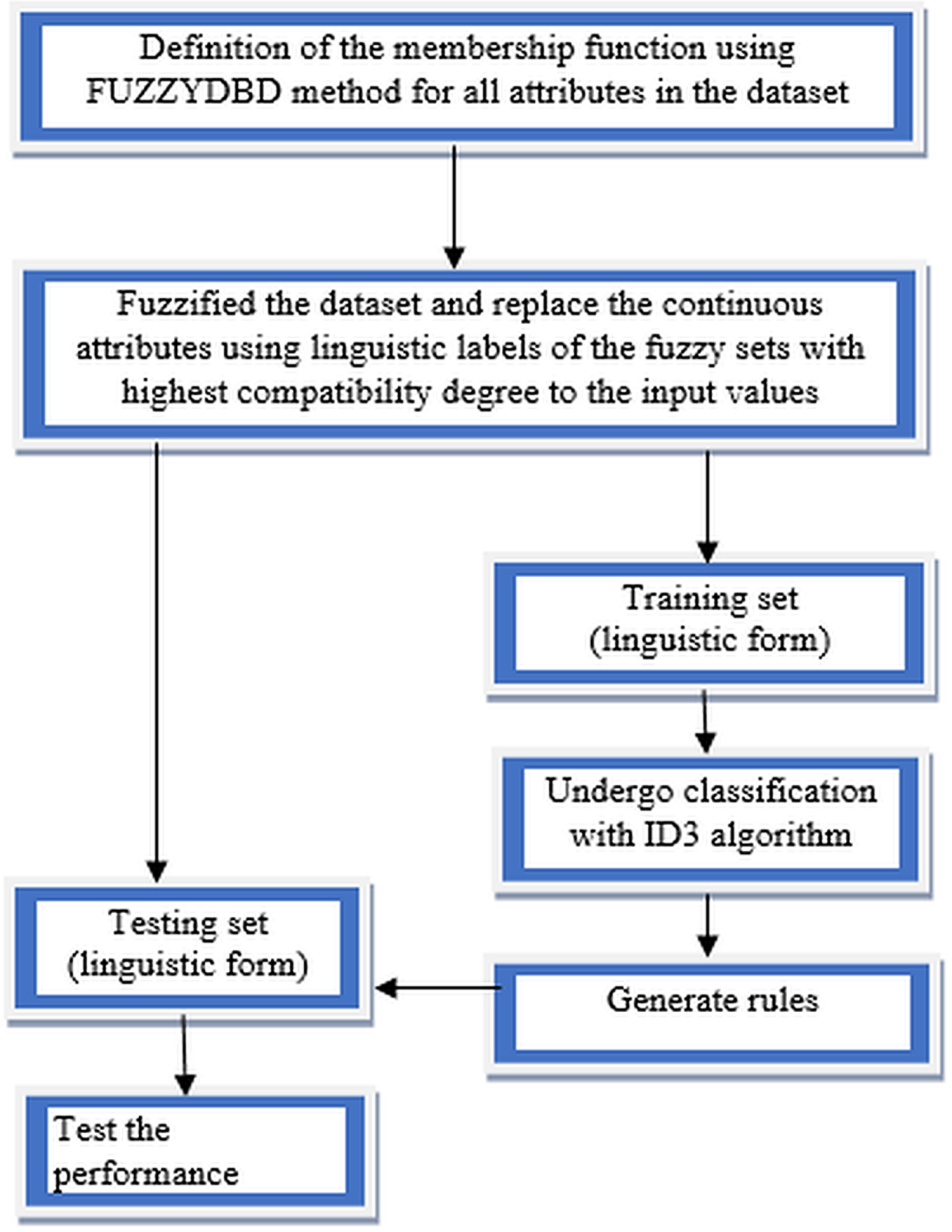
Flow of FID3 algorithm.

Fuzzy-ID3 algorithm implementation started with defining the membership functions using the FUZZYDBD method for all continuous attributes in the dataset. All the continuous attributes in each collected dataset would be defined with triangular equal partitioning membership functions and the standardised number of fuzzy sets (all attributes in the domain have the same count of fuzzy sets). The number of fuzzy sets that more commonly applied in the FUZZYDBD method is 3, 5 and 7 (Cintra, 2012). Then, the value of 3 also is the best-estimated number of fuzzy sets in the breast cancer domain, together with the value of 2 (Cintra, Camargo & Martin, 2009). Thus, in this research, all attributes in the collected datasets would adopt the value of 3 unless the medical expert indicates that the respective attribute might have a different number of fuzzy sets. A medical expert's assist is recommended in the development phase to obtain human interpretability as the suitability of the particular attributes’ variables impacts the classification process. Then, all the continuous attributes values in the dataset being fuzzified to generate fuzzy values of the data. The fuzzification is a process of conceptualisation that can reduce information overload in the decision-making task (Yuan & Shaw, 1995). Replacement of all the continuous attributes data using linguistic labels of the fuzzy sets with the highest compatibility degree to the input values or also known as fuzzy values is made to ensure the dataset in linguistic form. The discrete attributes (integer) also undergo the definition of membership functions using the FUZZYDBD method, fuzzification of data and replacement of linguistic labels processes to increase the accuracy results. Then, the split between training data and testing data was implemented after the whole dataset’s fuzzification process. After that, the training data will undergo classification with the ID3 algorithm, which is the chosen classifier to handle the fuzzified data. A fuzzy decision tree is generated using the fuzzified data, and the generated rules produce by the fuzzy decision tree will be used to test the effectiveness of the method. The most compatible rules can be directly used to classify the test data as both in linguistic forms. The classification rates of the method would be determined when tested with the testing set. The flow of FID3 algorithm has four vital steps as being illustrated in Fig. 4.

The process of data fuzzification in FID3 algorithm can occur where both fuzzy values (ex. low and medium) hit 0.5, an intermediate fuzzy value. Thus, the algorithm can randomly choose between the two linguistic labels or set up the standardisation of value. Nevertheless, the situation where both fuzzy values hit 0.5 that leads to unable to conduct the most accurate replacement of the linguistic variable very rarely occurs. FID3 algorithm's classification process retains the same computational technique with the ID3 algorithm that use Shannon’s entropy and information gain.

## Results

The experiments were conducted using three breast cancer datasets: WBCD (Original) dataset, WDBC (Diagnostic) dataset and Coimbra dataset. All the datasets are acquired from the UCI machine learning repository. Table 1 shows the brief descriptions of the collected breast cancer datasets. The Wisconsin Breast Cancer Database, known as WBCD (Original) dataset, is divided into two class attributes: benign and malignant. It contains nine predictive attributes: clump thickness, uniformity of cell size, uniformity of cell shape, marginal adhesion, single epithelial cell size, bland chromatin, bare nuclei, normal nucleoli and mitoses. The non-predictive attribute in this dataset is the ID number. The dataset consists of 699 instances, 458 benign samples and 241 malignant samples. It has 16 missing values, and listwise deletion was applied to handle the missing data, leading to 683 instances. The Breast Cancer Coimbra dataset used in this paper came from the Faculty of Medicine researchers at the University of Coimbra and University Hospital Centre of Coimbra. This dataset contains 116 instances and divided into two class attributes which are healthy controls and patients. This dataset also has nine predictive attributes such as age (years), BMI (kg/m^2^), glucose (mg/dL), insulin (µU/mL), HOMA, leptin (ng/mL), adiponectin (µg/mL), resistin (ng/mL) and MCP-1 (pg/dL).

**Table 1:** Details of the datasets applied in the experiment include the number of attributes, number of instances and classes.

Dataset	Number of attributes (including class attribute)	Number of instances	Number of classes
WBCD (original)	11	683	2
WDBC (diagnostic)	32	569	2
Coimbra	10	116	2

Lastly, this study applied Wisconsin Diagnostic Breast Cancer Dataset, also known as WDBC (Diagnostic). The source of this dataset is from the University of Wisconsin. This dataset comprises 569 instances with no missing values and has two class attributes: benign (B) or malignant (M). The dataset’s predictive attributes consist of ten-real valued features computed for each nucleus, such as radius, texture, perimeter, area, smoothness, compactness, concavity, concave points, symmetry, and fractal dimension. The mean, standard error and radius (mean of three largest values reading) are computed for each nucleus leading the dataset to have over 32 attributes, including the non-predictive attribute, patients’ ID and class attributes. For further explanation, field 3 stands for mean radius, field 13 stands for radius standard error, and field 23 stands for the worst radius (Oyelade et al., 2018).

The 10-fold cross-validation method is applied to test the effectiveness of the proposed method. The values of 3, 5 and 7 are more commonly implemented in the studies of the FUZZYDBD method, but the value of 3 also has lowest-error rates compared to other values when tested with breast cancer domain (Cintra, Camargo & Martin, 2009; Cintra et al., 2011b; Cintra & Neves, 2013; Cintra, 2012). Thus, for fuzzification, we defined all attributes in the datasets with three equally distributed triangular fuzzy sets (low, medium, high). The exception did for attributes radius standard error and worst radius in WDBC (Diagnostic) which will be defined with five triangular fuzzy sets (verylow, low, medium, high, veryhigh) which is the second most common value in the studies of FUZZYDBD method. Information and descriptions from the experts had normally been applied for the transformation of initial data (Zaitseva & Levashenko, 2016). The assist of domain experts is recommended in the modelling phase to obtain human interpretability (Seymoens et al., 2019). Linguistic values of radius standard error and worst radius were selected with the help of a medical expert. The increment in fuzzy sets value of radius standard error and worst radius determines the competence of mean radius results. The cell size is significant in determining the presence of cancer as an unhealthy cell always at the end spectrum, whether it is smaller or larger than a normal cell with an abnormal shape. Thus, an adequate number of linguistic variables for radius standard error and worst radius was needed to assess the mean radius results’ competency.

The confusion matrix is being implemented to acknowledge the capability of the proposed method in classification. The confusion matrix contains information about the actual classes and the predicted classes. From the confusion matrix information, accuracy, precision, recall and *F*1- the measure is being calculated. Meanwhile, the standard deviation of the accuracy also been calculated to determine the dispersion of the results. All the results were averaged over ten runs and implemented with the macro-averaged technique. The experimental results of breast cancer datasets are recorded in Table 2 in order to evaluate the performance of the proposed method.

**Table 2:** Experimental results of precision, recall, *F*1-measure and standard deviation (SD) when conducted with the collected datasets.

Dataset	Precision (%)	Recall (%)	*F*1-Measure (%)	SD
WBCD (original)	94.192	93.356	93.747	0.0050
WDBC (diagnostic)	94.294	94.000	94.137	0.0043
Coimbra	69.784	69.056	69.278	0.0116

The comparative analysis between the proposed method and other existing methods was performed to determine and verify the proposed model’s capability. Tables 3–5 show the comparison of accuracy between the proposed method and existing methods for each of the collected datasets. The existing methods used in the comparative analysis were support vector machine (SVM), C4.5 algorithm, naïve Bayes (NB), random forest (RF), K-Nearest Neighbour (KNN) and ID3 algorithm. The bold values in Tables 3–5 represent the highest accuracy.

**Table 3:** Comparison of accuracy between the proposed method and existing works using WBCD (Original) dataset.

Method	Accuracy (%)
SVM (Kumari, Singh & Ahlawat, 2019)	86.100
C4.5 algorithm (Saoud et al., 2019)	92.970
Naïve Bayes (Assiri, Nazir & Velastin, 2020)	91.810
Random forest (Pyingkodi et al., 2020)	91.660
KNN (Mushtaq et al., 2020)	92.570
ID3 algorithm	91.059
Proposed method	**94.362**

**Note:**

Values in bold represent the highest accuracy.

**Table 4:** Comparison of accuracy between the proposed method and existing works using WDBC (Diagnostic) dataset.

Method	Accuracy (%)
SVM (Chaurasia & Pal, 2020)	61.9614
C4.5 algorithm (Khan et al., 2017)	94.030
Naïve Bayes (Omondiagbe, Veeramani & Sidhu, 2019)	91.180
Random forest (Gondane & Susheela Devi, 2015)	89.370
KNN (Chaurasia & Pal, 2020)	92.7729
ID3 algorithm	–*
Proposed method	**94.534**

**Notes:**

* Result was not available as the method does not support the classification.

Values in bold represent the highest accuracy.

**Table 5:** Comparison of accuracy between the proposed method and existing works using Coimbra dataset.

Method	Accuracy (%)
SVM (Poorani & Balasubramanie, 2019)	65.960
C4.5 algorithm (Kayaalp & Basarslan, 2019)	68.000
Naïve Bayes (Fauziyyah, Abdullah & Nurrohmah, 2020)	67.700
Random forest (Austria et al., 2019)	70.310
KNN (Chiu, Li & Kuo, 2020)	67.120
ID3 algorithm	–*
Proposed method	**70.690**

**Notes:**

* Result was not available as the method does not support the classification.

Values in bold represent the highest accuracy.

**Figure 5:**
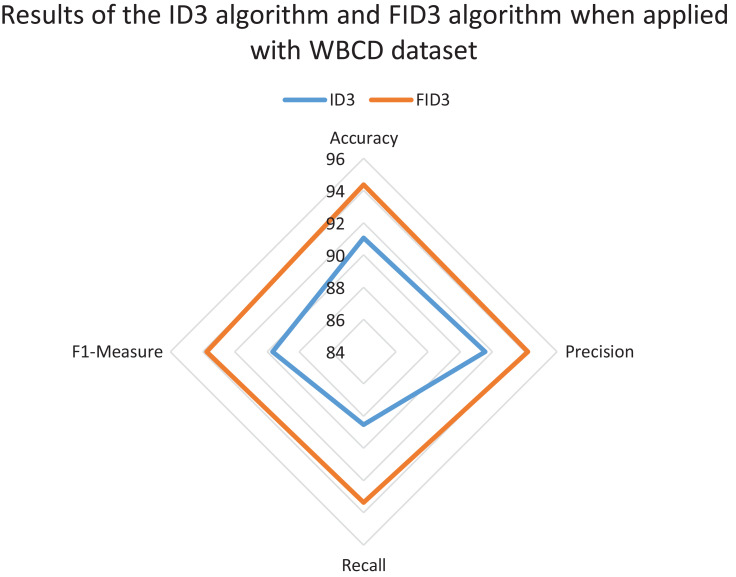
Results of the ID3 algorithm and FID3 algorithm when applied with the WBCD dataset.

The radar chart also was constructed to review and compare the overall classification performance of FID3 algorithm with existing ID3 algorithm. The reviewed between the two methods was carried out to identify whether FID3 algorithm managed to outperform ID3 algorithm. ID3 algorithm is incapable of conducting the classification process for the WDBC dataset and the Coimbra dataset as both datasets consisting of real-valued data. Thus, the comparison of results between the two methods only conducted using the WBCD dataset. The ID3 algorithm classification process is possible with the WBCD dataset because it mainly consists of small integer attributes, ranging between one to ten. ID3 algorithm would treat the attributes in WBCD dataset as categorical data. Figure 5 shows the ID3 algorithm and FID3 algorithm results when applied with WBCD dataset. The overall performance of FID3 algorithm is better than the ID3 algorithm as all the plotting points defined as accuracy, precision, recall and *F*1-measure in the chart have longer radii.

A statistical test known as *t*-test was implemented to determine whether the classification accuracy of traditional ID3 algorithm and FID3 algorithm is statistically different. The *t*-value and *p*-value were identified using the accuracy results of 10 independent runs which the degree of freedom equal to nine (*n* − 1). The significance level (α) of the *t*-test was set as 0.05, and a two-tailed test was applied. If the *p*-value is greater than α, then the null hypothesis, H0, stated that no significant difference between the ID3 algorithm and FID3 algorithm would be accepted. Otherwise, if the *p*-value lower than α, then the alternative hypothesis, H1, stated that a significant difference would be accepted. In *t*-test of independent means for WBCD dataset, the *t*-value is 18.48666 while the *p*-value is <0.00001. The result is significant at *p* < 0.05. According to the study, *p*-values were lower than the significance level. Thus, the null hypothesis, H0 is rejected and H1, the alternative hypothesis is accepted where the result is significant at *p*-value < 0.05. The accuracy of FID3 algorithm significantly increases compared to the ID3 algorithm.

## Discussion

Fuzzy- ID3 algorithm acquired an accuracy of 94.362%, which is the best accuracy result in the comparative analysis when tested with WBCD dataset. The accuracy of FID3 algorithm higher than C4.5 algorithm, the second-best method by 1.392% and KNN, the third-best method by 1.792%. The proposed method had achieved higher accuracy than Fuzzy GAP, the hybrid genetic programming-genetic algorithm that develops a fuzzy classifier for each class by searching for a tree that got an accuracy of 92.53% (Orriols-Puig, Casillas & Bernadó-Mansilla, 2009). Then, the FID3 algorithm obtained the classification accuracy of 94.534% when implemented with WDBC dataset, which was better than the C4.5 algorithm, KNN, NB, RF and SVM. The accuracy of FID3 algorithm is higher by 0.504% than C4.5 algorithm, which is the second-best method and KNN, the third-best method by 1.7611%. Based on the finding, the result had surpassed PAM LOR V.2.0, clustering data mining technique by Badiang, Gerardo & Medina (2019) that only obtain an accuracy of 88.75% when applied with WDBC dataset. FID3 algorithm had outperforms FUZZY ID3-L-WABL, an improved version of the classic FUZZY ID3 algorithm by Kantarci-Savas & Nasibov (2017) that obtains accuracy of only 90.87% when implemented with WDBC dataset. This method obtained higher accuracy than Fuzzy GAP that got only 90.49% with this dataset (Orriols-Puig, Casillas & Bernadó-Mansilla, 2009). Lastly, FID3 algorithm managed to acquire accuracy of 70.69%, which is significantly higher than RF, the second-best method that obtained only 70.31% when tested with Coimbra dataset. This result outdoes PAM LOR V.2.0 by Badiang, Gerardo & Medina (2019) that only obtain 55.17% when applied with Coimbra dataset. The results conveyed that the proposed method has better performance and comparable to many existing works. Based on the statistical test executed, the accuracy of FID3 algorithm significantly increases compared to the ID3 algorithm where the result is significant at *p*-value < 0.05. Overall, the FID3 algorithm is more effective than traditional ID3 algorithm in solving classification problems and generates better accuracy, precision, recall and *F*1-measure. FID3 algorithms also manage to overcome the limitation of the ID3 algorithm that incapable of handling continuous-valued data. ID3 algorithm treats continuous attributes as discrete or categorical attributes with many possible values which would arouse problems, especially in the classification of real-valued data.

FUZZYDBD method comprises of the Wang Mendel method and Equalised Universe Method that was widely used in literature and had shown excellent performance when tested with breast cancer dataset. The implementation of FUZZYDBD method in the fuzzy-ID3 algorithm is used to set up the fuzzy sets parameters in order to increase the suitability and effectiveness of data fuzzification, especially in breast cancer domain. FID3 algorithm undergoes both fuzzification of data and the replacement process of continuous-valued attributes with the linguistic variable of fuzzy sets that has the highest compatibility degree. These processes allow FID3 algorithm to handle any data type and overcome the limitation of the ID3 algorithm. The algorithm also generates a decision tree with lower depth and fewer branches. The existing study identified that a low complexity decision tree would have lower variance, thus better predictive accuracy (Olaru & Wehenkel, 2003). FID3 algorithm uses logical reasoning like a traditional ID3 algorithm. The application of a single process of data fuzzification in the FID3 algorithm allows the decision tree in each fold to select the best rules for the new instances directly as both training, and testing sets are in the same formation (linguistic form). The decision tree makes a deduction from the model’s generated rules as it will choose the class of the rules with the highest compatibility degree or is most compatible with the testing data.

Nevertheless, there are limitations in the proposed method because it still has the characteristics of traditional decision tree-like having a high variance, tendency to overfit and instability. FID3 algorithm having multi-value bias problem despite FUZZYDBD setting up standardisation number of terms in attributes. The problem happens because the attributes’ terms and elements would reduce as classification started. There are stark differences in accuracy performance between the datasets because of the small sample size limitation, especially in Coimbra dataset (Patrício et al., 2018). Overfitting is hard to avoid, and despite a cross-validation technique performed to minimise bias, but it is not possible to entirely eliminate it. The Coimbra dataset also contains noise leading to poor classification result (De Brito, 2018). Theoretically, FID3 algorithm still retains the same properties of the traditional ID3 algorithm but more robust. In traditional ID3 algorithm or other decision tree learning, even the differences of 0.01 continuous values in the data would lead different pathway and classes in the tree, but FID3 algorithm taking account the membership degree of the particular input and has a high tolerance to data uncertainty.

## Conclusion

Fuzzy-ID3 algorithm is reliable and managed to generate good performances in the classification of breast cancer data. Implementation of FUZZYDBD method as an automatic fuzzy database definition method in the fuzzification process of the fuzzy decision tree is compelling, consistent, and straightforward, allowing the fast fuzzification process to occur. The proposed method resolves the drawback of traditional ID3 algorithm of incapable of handling continuous-valued data and has higher accuracy results. The proposed method of FID3 algorithm also has lower complexity, easy to understand and high interpretability compare to other fuzzy decision tree methods as all the steps in FID3 algorithm is more straightforward. The proposed method’s limitations, such as instability and overfitting issues can be resolved through future works like implementing ensemble methods. Attribute related methods also can be applied for better attribute selection criteria in FID3 algorithm. Overall, the implementation of FID3 algorithm with FUZZYDBD method is useful and productive in the classification of data.

## Supplemental Information

10.7717/peerj-cs.427/supp-1 Supplemental Information 1 Code for Coimbra dataset. To run the FID3 algorithm-FUZZYDBD:
1. Open id310fold.py (python language)
2. Make sure the dataset is w5.data/WBCDF1.data/coimbrafold5.data
(according to the folder)
id310fold.py is located in the FID3 file. Apply the w5.data/WBCDF1.data/coimbrafold5.data dataset located in the same FID3 file as it is the latest data that has already undergone data transformation into the linguistic format. All the instances have been shuffled well.

10.7717/peerj-cs.427/supp-2 Supplemental Information 2 Code for WBCD dataset. To run the FID3 algorithm-FUZZYDBD:
1. Open id310fold.py (python language)
2. Make sure the dataset is w5.data/WBCDF1.data/coimbrafold5.data
(according to the folder)
id310fold.py is located in the FID3 file. Apply the w5.data/WBCDF1.data/coimbrafold5.data dataset located in the same FID3 file as it is the latest data that has already undergone data transformation into the linguistic format. All the instances have been shuffled well.

10.7717/peerj-cs.427/supp-3 Supplemental Information 3 Code for WDBC dataset. To run the FID3 algorithm-FUZZYDBD:
1. Open id310fold.py (python language)
2. Make sure the dataset is w5.data/WBCDF1.data/coimbrafold5.data
(according to the folder)
id310fold.py is located in the FID3 file. Apply the w5.data/WBCDF1.data/coimbrafold5.data dataset located in the same FID3 file as it is the latest data that has already undergone data transformation into the linguistic format. All the instances have been shuffled well.

## Additional Information and Declarations

### Data Availability

The following information was supplied regarding data availability: Raw data are available in the Supplemental Files.
